# Mg-MOF-74/MgF_2_ Composite Coating for Improving the Properties of Magnesium Alloy Implants: Hydrophilicity and Corrosion Resistance

**DOI:** 10.3390/ma11030396

**Published:** 2018-03-07

**Authors:** Wei Liu, Zhijie Yan, Xiaolu Ma, Tie Geng, Haihong Wu, Zhongyue Li

**Affiliations:** 1 School of Mechanical & Electrical Engineering, Henan University of Technology, Zhengzhou 450001, China; weiliuhaut@gmail.com (W.L.); yanzhijiehaut@gmail.com (Z.Y.); maxiaolu@haut.edu.cn (X.M.); gengtiehaut@gmail.com (T.G.); hhwu@haut.edu.cn (H.W.); 2 School of Materials Science and Engineering, Henan Polytechnic University, Jiaozuo 454000, China

**Keywords:** magnesium alloys, corrosion resistance, hydrophilicity, metal organic framework, composite coating

## Abstract

Surface modification on Mg alloys is highly promising for their application in the field of bone repair. In this study, a new metal–organic framework/MgF_2_ (Mg-MOF-74/MgF_2_) composite coating was prepared on the surface of AZ31B Mg alloy via pre-treatment of hydrofluoric acid and in situ hydrothermal synthesis methods. The surface topography of the composite coating is compact and homogeneous, and Mg-MOF-74 has good crystallinity. The corrosion resistance of this composite coating was investigated through Tafel polarization test and immersion test in simulated body fluid at 37 °C. It was found that Mg-MOF-74/MgF_2_ composite coating significantly slowed down the corrosion rate of Mg alloy. Additionally, Mg-MOF-74/MgF_2_ composite coating expresses super-hydrophilicity with the water contact angle of nearly 0°. In conclusion, on the basis of MgF_2_ anticorrosive coating, the introduction of Mg-MOF-74 further improves the biological property of Mg alloys. At last, we propose that the hydrophilicity of the composite coating is mainly owing to the large number of hydroxyl groups, the high specific surface area of Mg-MOF-74, and the rough coating produced by Mg-MOF-74 particles. Hence, Mg-MOF-74 has a great advantage in enhancing the hydrophilicity of Mg alloy surface.

## 1. Introduction

Magnesium (Mg) alloys have drawn interest as biomaterials to repair bone defects in recent years [1,2,3,4,5]. Compared to other biomaterials, such as titanium alloys and stainless steel, Mg alloys have a lot of advantages: (i) Mg alloys are much closer to natural bone in density and elastic modulus, eliminating the stress shielding effects [6,7,8]; (ii) Mg^2+^ is essential to human beings [9]; (iii) because of degradability of Mg in the human body, implants made of Mg alloys will not require a secondary removal surgery [10]. However, their disadvantage as implants is their poor corrosion resistance, greatly hindering their widespread clinical application so far.

Surface modification of Mg alloys using a biocompatible coating has shown to be a promising method, which can lower the corrosion rate, and also improve the interaction between implant and tissue [11]. Thus far, a variety of materials—such as bioactive ceramics [12], bioinert ceramics [5], MgF_2_ coating [13], anode oxide film [14], biodegradable polymers [4,15], etc.—have been used as a coating material. Among them, MgF_2_ coating is a common one because of simple process, low cost, and good corrosion resistance [16,17,18,19]. Recently, considerable efforts have been made to prepare composite coating of MgF_2_ and other materials for better property. Ren et al. synthesized a double layered composite coating of calcium phosphate glass/MgF_2_ on AZ31 substrate by pre-treatment of hydrofluoric acid and sol-gel dip coating method, showing the improved corrosion resistance [20]. Feng et al. prepared PRC-HA/MgF_2_ coating on Mg-Zn-Ca alloy by pulse reverse current electrodeposition based on MgF_2_ coating, improving the corrosion resistance and the bioactivity [21]. However, a hydrophilic surface is believed to be important for bone repair biomaterials, it can improve the biocompatibility of implant in blood [22], and it is also beneficial for bone cells adhesion and proliferation to promote further bone growth [23,24]. There have been many studies on hydrophilicity surface of titanium alloys [25,26,27], but the study of hydrophilicity on magnesium alloys is relatively less [28]. So far, few studies have been published about the hydrophilicity coating of Mg alloys based on MgF_2_.

Metal–organic frameworks (MOFs), comprising of metal ions and various organic ligands, have been used over past several years in many fields, such as gas separation [29], drug delivery systems [30,31,32], corrosion protection of metals [33,34], and so on. Due to the excellent characteristics of MOFs with high specific surface area and various functional groups, some MOFs are especially biocompatible, it is possible to introduce MOF as the hydrophilicity coating.

The purpose of this study is to improve both hydrophilicity and corrosion resistance of Mg alloys intended as implant. Here, a new Mg-MOF-74/MgF_2_ composite coating was fabricated by pre-treatment of hydrofluoric acid and hydrothermal in situ growth methods. Mg-MOF-74 is a classic biocompatible MOF material constructed with Mg^2+^ ion and 2,5-dihydroxyterephthalic [35]. It is selected here because: (i) Mg-MOF-74 contains the same metal Mg with MgF_2_ and Mg alloy, avoiding the introduction of other disadvantageous metals and being helpful to the formation of compact composite coating as well; (ii) a large number of hydroxyl groups on the outer and pore surface of Mg-MOF-74 might be beneficial to improve hydrophilicity; (iii) it has big surface area, good biocompatibility, and stability. Hydrophilicity was investigated through water contact angle testing, and the mechanism was discussed. The study on corrosion resistance was performed using an electrochemical test and the H_2_ gassing experiment in simulated body fluid (SBF) at physiological temperature, respectively.

## 2. Materials and Methods 

### 2.1. Reagents and Materials

AZ31B Mg alloy (composition: 2.5–3.5 Al, 0.6–1.4 Zn, 0.2–1.0 Mn, 0.04 Ca, 0.08 Si, 0.003 Fe, 0.001 Ni, 0.01 Cu, Mg, all in wt. %) was purchased from Luoyang Shengte Metalware Co., Ltd. (Luoyang, China). 2,5-dihydroxyterephthalic acid (98%), sodium hydrogen carbonate (99.8%), potassium chloride (99.5%), di-potassium hydrogen phosphate trihydrate (99%), magnesium chloride hexahydrate (98%), calcium chloride (96%), sodium sulfate (99%), and tris-hydroxymethyl aminomethane (AR) were bought from the Shanghai Macklin Biochemical Technology Co., Ltd. (Shanghai, China). Magnesium nitrate hexahydrate (99%) and anhydrous methanol were bought from the Tianjin Hengxing Chemical Reagent co., Ltd. (Tianjin, China). Ethanol and sodium chloride (99.5%) were bought from the Tianjin Chemical Reagent Supply and Marketing Company (Tianjin, China). Hydrofluoric acid (40%) was bought from the Luoyang Haohua Chemical Reagent Co., Ltd. (Luoyang, China). *N*,*N*-dimethylformamide (99.5%) was bought from the Sinopharm Chemical Reagent Co., Ltd. (Shanghai, China). Hydrochloric acid (36–38%) was purchased from the Zhengzhou Paini Chemical Reagent Factory (Zhengzhou, China).

### 2.2. Instrumentation

The phases of the samples were analyzed on a Bruker D8 Advance X-ray diffractometer (Cu-Kα radiation) with the scan speed and step of 10°⋅min^−1^ and 0.02°, respectively. The surface, cross-sectional morphologies and elemental composition of the coating were observed by scanning electron microscopy (SEM, FEI INSPECT F50, Hillsboro, OR, USA) equipped with energy dispersive X-ray spectrometer (EDS, BRUKER QUANTAX 400, Karlsruhe, Germany). Electrochemical tests were carried out using an electrochemical workstation (RST5000, Zhengzhou Shiruisi Inc., Zhengzhou, China) with a three-electrode system. In immersion testing, a simple setup was made by ourselves to record the evolved hydrogen volumes [36]. The static water contact angles were measured by using a contact angle meter (JC2000D1, Shanghai Zhongchen Inc., Shanghai, China) to examine surface wettability of all samples.

### 2.3. Sample Preparation 

Pretreatment of Mg Alloy: AZ31B Mg alloy was cut into 20 × 20 × 2 mm sheets as substrates. Then Mg alloy sheet was ground sequentially with 240^#^, 600^#^, and 1000^#^ silicon carbide paper. Next, the sheet was rinsed ultrasonically in ethanol for 15 minutes. At last, it was washed with deionized water and dried in air.

Preparation of MgF_2_ coating: To obtain the MgF_2_ coating, the treated substrate was immersed in 40% (*v*/*v*) hydrofluoric acid at room temperature for 24 h. The specimen was later rinsed in deionized water and dried in the air.

**Figure 1:**
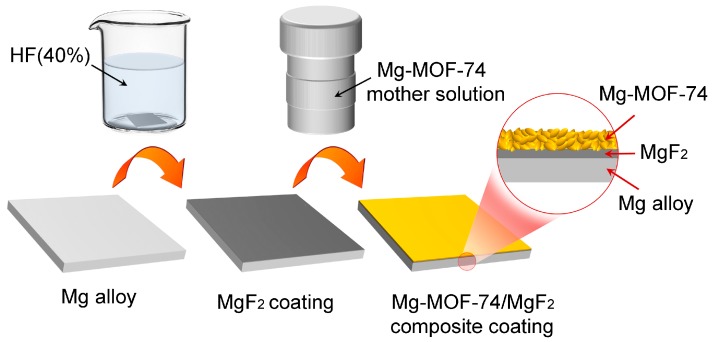
Schematic diagram of the preparation process of Mg-MOF-74/MgF_2_ composite coating.

Fabrication of Mg-MOF-74/MgF_2_ composite coating: Mg-MOF-74 coating was provided by hydrothermal synthesis method [37], and the preparation of reaction solution is as follows. Magnesium nitrate hyxahydrate (2.5 mmoL) and 2,5-dihydroxyterephthalic acid (0.8 mmoL) were dissolved in a solvent mixture of *N*,*N*-dimethylformamide (60 mL), ethanol (4 mL) and deionized water (4 mL). The solution was transferred to an autoclave. Then specimen above was dipped vertically into the solution in an oven for 24 h at 125 °C to yield Mg-MOF-74 crystals on the MgF_2_ coating. The process of purification and activation is as follows: the specimen with Mg-MOF-74/MgF_2_ composite coating was steeped in anhydrous methanol for 12 h, then this sample was treated under vacuum at 100 °C for 3 h to remove the solvent molecules from the pores of Mg-MOF-74. The process on fabrication of Mg-MOF-74/MgF_2_ composite coating is illustrated schematically in Figure 1.

### 2.4. Corrosion Resistance Study

Tafel polarization tests: A three-electrode system was employed, where the samples (with 1 cm^2^ exposed area), saturated calomel electrode (SCE), and a platinum wire served as the working electrode (WE), reference electrode (RE), and counter electrode (CE), respectively. The solution was SBF at 37 °C, which was made up according to previous studies [38], and the information about reagents used can be found in Section 2.1 above. Tafel polarization tests were performed at a scan rate of 0.5 Mv·s^−1^, and the samples were immersed in the SBF solution for about 30 min to obtain an electrochemical steady state before the tests.

Immersion tests: The samples were immersed separately in SBF solution controlled with a water bath at 37 °C. The SBF volume to the exposed area of the sample was 100 mL·cm^−2^. The volumes of hydrogen produced were recorded at different times to investigate the corrosion resistance of samples.

### 2.5. Hydrophilicity Study

In the process of water contact angle measurements, 1 µL of water was dropped on the surface of samples, and then the images of the droplet were captured for 5 s after dropping. Three spots were chosen at random on each sample surface to make the measurement and the results obtained were averaged.

## 3. Results and Discussion

### 3.1. Microstructure and Composition of Coating

**Figure 2:**
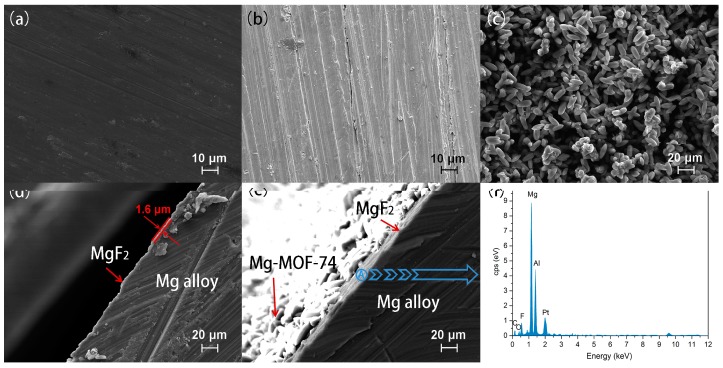
SEM images. The vertical view of (**a**) bare Mg alloy, (**b**) MgF_2_-coated Mg alloy, and (**c**) Mg-MOF-74/MgF_2_-coated Mg alloy; the cross-sectional view of (**d**) MgF_2_-coated Mg alloy and (**e**) Mg-MOF-74/MgF_2_-coated Mg alloy; (**f**) the EDS spectrum on MgF_2_ middle layer.

Figure 2a–c depicts the surface morphologies of uncoated, MgF_2_-coated and Mg-MOF-74/MgF_2_-coated Mg alloys. As shown in Figure 2b, the scratch caused by the grinding is still obvious after hydrofluoric acid etching, because the MgF_2_ layer is very thin. It also can be seen from the cross-sectional micrograph of Mg alloy substrates with MgF_2_ coating (Figure 2d). Moreover, a dense intergrown Mg-MOF-74 crystal layer is observed on the MgF_2_ coating (Figure 2c,e). It can be attributed to that Mg source in MgF_2_ could provide activated sites to connect with organic ligands, while Mg source in reaction solution is employed for growth of Mg-MOF-74 coating as well, named ‘dual-Mg-source’ method. Notably, this method not only benefits the coating compactness, but also enhances the binding force between coating and substrate [39,40]. The EDS spectrum on MgF_2_ middle layer (area circled in Figure 2e) shown in Figure 2f displays the existence of fluoride, suggesting that MgF_2_ still existed after Mg-MOF-74 was coated onto it.

**Figure 3:**
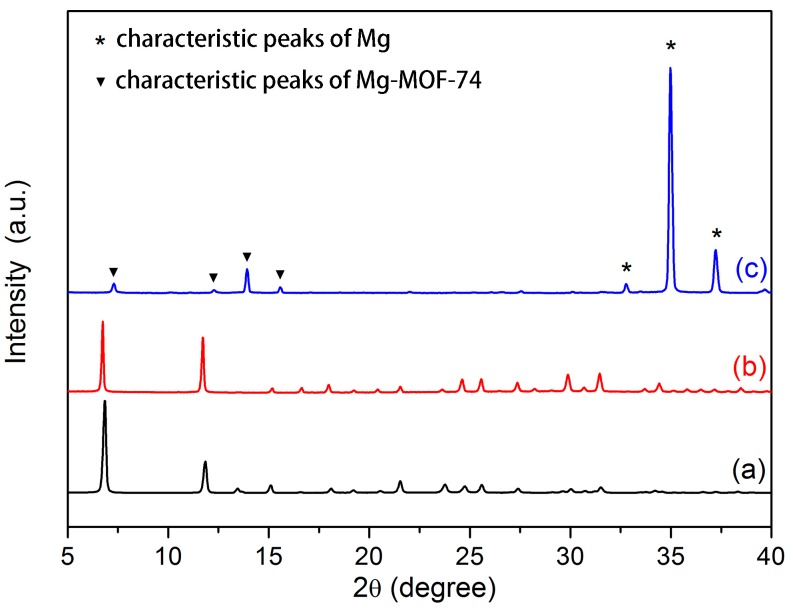
XRD patterns of (**a**) Mg-MOF-74 powder; (**b**) Mg-MOF-74 powder immersed in SBF for seven days; (**c**) Mg-MOF-74/MgF2 coated Mg alloy.

Figure 3a depicts the X-ray diffraction (XRD) pattern of the Mg-MOF-74 powder collected at the bottom of autoclave, produced in the process of Mg-MOF-74 coating preparation. The peaks at 7.5° and 12° match well with the characteristic peaks of Mg-MOF-74 in literature [41]. The XRD pattern of Mg-MOF-74 powder almost does not change after seven days of immersion in SBF (Figure 3b). It suggests that Mg-MOF-74 is stable enough in SBF. As can be seen in Figure 3c, the pattern of the Mg-MOF-74/MgF_2_ coated Mg alloy agrees with the Mg-MOF-74 powder and Mg (marked with *, JCPDS No. 65-3365), indicating that the outer coating consisted mainly of crystalline Mg-MOF-74. However, the characteristic peaks of Mg-MOF-74 in Mg-MOF-74/MgF_2_ coating become weaker and shift slightly to the right compared to the powder sample. It is most likely due to the thin crystal film formation of Mg-MOF-74.

### 3.2. Corrosion Resistance Property

**Figure 4:**
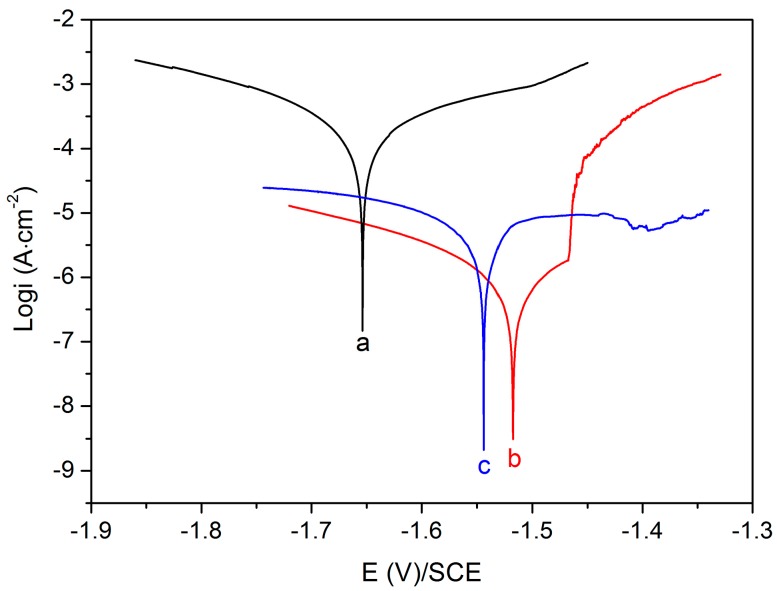
Tafel polarization curve of (**a**) uncoated Mg alloy; (**b**) MgF_2_-coated Mg alloy; and (**c**) Mg-MOF-74/MgF_2_-coated Mg alloy in SBF.

Electrochemical test: Tafel polarization test is a common electrochemical test used to investigate the corrosion resistance property of Mg alloy. The high corrosion potential (E_corr_) and low corrosion current density (i_corr_) indicate the good corrosion resistance [42]. Figure 4 shows the Tafel polarization curves of different samples in SBF solution at 37 °C, and the E_corr_ and i_corr_ obtained are summarized in Table 1. The E_corr_ values of the MgF_2_-coated and Mg-MOF-74/MgF_2_-coated Mg alloys are −1.52 V and −1.54 V, which are higher than that of the uncoated Mg alloy (−1.65 V). These two coated Mg alloys present two orders of magnitude reduction on i_corr_ values (1.19 × 10^−6^ and 6.46 × 10^−6^ A·cm^−2^) compared to the uncoated one (2.18 × 10^−4^ A·cm^−2^). It indicates that the MgF_2_ and Mg-MOF-74/MgF_2_ composite coatings could obviously enhance the corrosion resistance of Mg alloys.

**Figure 5:**
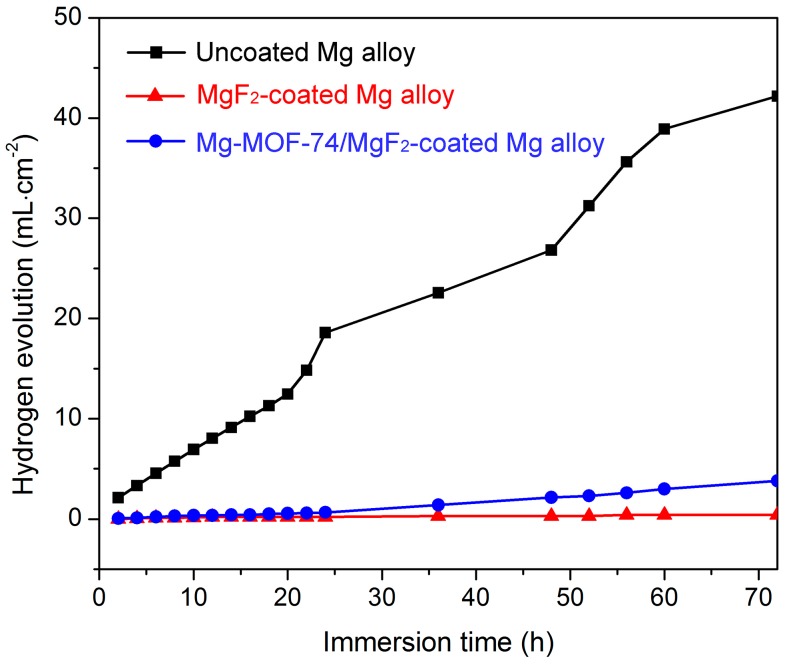
Volume of hydrogen gas released as a function of immersion time in SBF for uncoated, MgF_2_-coated, and Mg-MOF-74/MgF_2_-coated Mg alloy.

Immersion test: H_2_ will be produced when Mg alloy is in corrosive medium, and the larger the hydrogen volume is, the more serious the corrosion [4]. Herein, immersion test was carried out to further evaluate the corrosion resistance of the composite coating. Figure 5 shows the hydrogen evolution results of the different specimens in SBF for 72 h. Hydrogen releases from the Mg-MOF-74/MgF_2_-coated and MgF_2_-coated Mg alloy much slower than from the uncoated one. The uncoated Mg alloy releases a far bigger amount of hydrogen than the Mg-MOF-74/MgF_2_-coated and MgF_2_-coated Mg alloys in 72 h. It proves that both MgF_2_ layer and Mg-MOF-74/MgF_2_ composite coating can protect effectively Mg alloy.

The results of these two tests prove that Mg-MOF-74/MgF_2_ composite coating could obviously enhance the corrosion resistance of Mg alloy, although its anticorrosion effect is slightly worse than that of single MgF_2_ coating. It is potentially due to partly dissolution of MgF_2_ layer during the Mg-MOF-74 synthesis, leading to greater probability of pit corrosion.

### 3.3. Hydrophilicity

**Figure 6:**
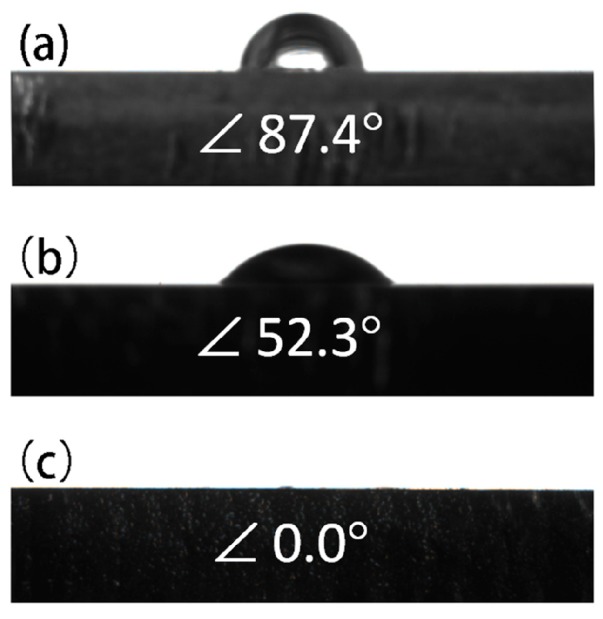
Photograph of water droplet on the surface of (**a**) uncoated Mg alloy; (**b**) MgF_2_-coated Mg alloy; (**c**) Mg-MOF-74/MgF_2_-coated Mg alloy.

The good hydrophilicity surface of bone repair implants is beneficial for bone cells adhesion and proliferation, promoting further bone growth. The water contact angle is often used to evaluate the hydrophilicity of material surface. Moreover, the smaller the contact angle is, the better the hydrophilicity [43]. Figure 6 shows the photographs of water droplets on the surfaces of uncoated, MgF_2_-coated and Mg-MOF-74/MgF_2_-coated Mg alloy samples. The surface wettability of Mg alloy is obviously changed after being coated. The contact angles of the uncoated, MgF_2_, and Mg-MOF-74/MgF_2_ coated samples are around 87.4°, 52.3°, and 0°, respectively, indicating the outer Mg-MOF-74 coating is superhydrophilic. According to previous study [44], hydrophilicity is connected with chemical constitution, microstructure and roughness. The superhydrophilicity of Mg-MOF-74 can be attributed to the points as follows: (i) the massive hydroxyl groups exposed on the pore surface of Mg-MOF-74, which can form hydrogen bond with water; (ii) the high specific surface area of Mg-MOF-74 as a MOF material [45]. According to the Wenzel model [44], a high specific surface area leads to excellent hydrophilicity; (iii) the rough coating produced by Mg-MOF-74 particles could decrease the contact angle between the water drop and the solid surface. Therefore, Mg-MOF-74 has great advantage to increase the hydrophilicity of Mg alloy surface.

## 4. Conclusions

In this work, the Mg-MOF-74/MgF_2_ composite coating is fabricated on the AZ31B Mg alloy surface by pre-treatment of hydrofluoric acid and hydrothermal in situ growth methods. This coating has greatly improved the anti-corrosion and hydrophilicity of Mg alloys. In this composite coating, the role of MgF_2_ is as an anticorrosion coating to lower degradation rate. Mg-MOF-74 mainly plays a part in improving the hydrophilicity, which is probably conducive to bone cell adhesion and proliferation on Mg alloys. Furthermore, the mechanism has been discussed and we propose that the massive hydroxyl groups exposed on the pore surface, the high specific surface area of Mg-MOF-74, and the rough coating produced by Mg-MOF-74 particles contribute mainly to improve hydrophilicity of the coating. Especially, this work would offer a thought for the application of MOFs to bone tissue engineering.

## Figures and Tables

**Table 1:** Results of Tafel polarization tests in SBF

Sample	E_corr_ (V vs. SCE)	i_corr_ (A·cm^−2^)
Uncoated Mg alloy	−1.65	2.18 × 10^−4^
MgF_2_-coated Mg alloy	−1.52	1.19 × 10^−6^
Mg-MOF-74/MgF_2_-coated Mg alloy	−1.54	6.46 × 10^−6^

## References

[1] Waizy H., Seitz J.M., Reifenrath J., Weizbauer A., Bach F.W., Meyer-Lindenberg A., Denkena B., Windhagen H. (2013). Biodegradable magnesium implants for orthopedic applications. J. Mater. Sci..

[2] Witte F. (2010). The history of biodegradable magnesium implants: A review. Acta Biomater..

[3] Razavi M., Fathi M., Savabi O., Razavi S.M., Heidari F., Manshaei M., Vashaee D., Tayebi L. (2014). In vivo study of nanostructured diopside (CaMgSi_2_O_6_) coating on magnesium alloy as biodegradable orthopedic implants. Appl. Surf. Sci..

[4] Shi P., Niu B., Shanshan E., Chen Y., Li Q. (2015). Preparation and characterization of PLA coating and PLA/MAO composite coatings on AZ31 magnesium alloy for improvement of corrosion resistance. Surf. Coat. Technol..

[5] Córdoba L.C., Montemor M.F., Coradin T. (2016). Silane/TiO_2_ coating to control the corrosion rate of magnesium alloys in simulated body fluid. Corros. Sci..

[6] Staiger M.P., Pietak A.M., Huadmai J., Dias G. (2006). Magnesium and its alloys as orthopedic biomaterials: A review. Biomaterials.

[7] Xin Y., Hu T., Chu P.K. (2011). In vitro studies of biomedical magnesium alloys in a simulated physiological environment: A review. Acta Biomater..

[8] Yang L., Zhang E. (2009). Biocorrosion behavior of magnesium alloy in different simulated fluids for biomedical application. Mater. Sci. Eng. C.

[9] Song G., Song S. (2007). A possible biodegradable magnesium implant material. Adv. Eng. Mater..

[10] Saris N.L., Mervaala E., Karppanen H., Khawaja J.A., Lewenstam A. (2000). Magnesium: An update on physiological, clinical and analytical aspects. Clin. Chim. Acta.

[11] Wang J., Tang J., Zhang P., Li Y., Wang J., Lai Y., Qin L. (2012). Surface modification of magnesium alloys developed for bioabsorbable orthopedic implants: A general review. J. Biomed. Mater. Res. B Appl. Biomater..

[12] Zhang X., Li Q., Li L., Zhang P., Wang Z., Chen F. (2012). Fabrication of hydroxyapatite/stearic acid composite coating and corrosion behavior of coated magnesium alloy. Mater. Lett..

[13] Jiang H., Wang J., Chen M., Liu D. (2017). Biological activity evaluation of magnesium fluoride coated Mg-Zn-Zr alloy in vivo. Mater. Sci. Eng. C.

[14] Li B., Han Y., Qi K. (2014). Formation Mechanism, Degradation Behavior, and Cytocompatibility of a Nanorod-Shaped HA and Pore-Sealed MgO Bilayer Coating on Magnesium. ACS Appl. Mater. Interfaces.

[15] Jin W., Hao Q., Peng X., Chu P.K. (2016). Enhanced corrosion resistance and biocompatibility of PMMA-coated ZK60 magnesium alloy. Mater. Lett..

[16] Makar G.L., Kruger J. (1993). Corrosion of magnesium. Int. Mater. Rev..

[17] Thomann M., Krause C., Angrisani N., Bormann D., Hassel T., Windhagen H., Meyer-Lindenberg A. (2010). Influence of a magnesium-fluoride coating of magnesium-based implants (MgCa0.8) on degradation in a rabbit model. J. Biomed. Mater. Res. A.

[18] Drynda A., Seibt J., Hassel T., Bach F.W., Peuster M. (2013). Biocompatibility of fluoride-coated magnesium-calcium alloys with optimized degradation kinetics in a subcutaneous mouse model. J. Biomed. Mater. Res. A.

[19] Su Y., Lu Y., Su Y., Hu J., Lian J., Li G. (2015). Enhancing the corrosion resistance and surface bioactivity of a calcium-phosphate coating on a biodegradable AZ60 magnesium alloy via a simple fluorine post-treatment method. RSC Adv..

[20] Ren M., Cai S., Liu T., Huang K., Wang X., Zhao H., Niu S., Zhang R., Wu X. (2014). Calcium phosphate glass/MgF_2_ double layered composite coating for improving the corrosion resistance of magnesium alloy. J. Alloys Compd..

[21] Feng Y., Zhu S., Wang L., Chang L., Yan B., Song X., Guan S. (2017). Characterization and corrosion property of nano-rod-like HA on fluoride coating supported on Mg-Zn-Ca alloy. Bioact. Mater..

[22] Zhang Y., Forsyth M., Hinton B.R.W. (2014). The Effect of Treatment Temperature on Corrosion Resistance and Hydrophilicity of an lonic Liquid Coating for Mg-Based Stents. ACS Appl. Mater. Interfaces.

[23] Lang N.P., Salvi G.E., Huyng-Ba G., Ivanovski S., Donos N., Bosshardt D.D. (2011). Early osseointegration to hydrophilic and hydrophobic implant surfaces in humans. Clin. Oral Implants Res..

[24] Ye X., Cai S., Xu G., Dou Y., Hu H. (2012). Synthesis of mesoporous hydroxyapatite thin films using F127 as templates for biomedical applications. Mater. Lett..

[25] Rupp F., Scheideler L., Olshanska N., de Wild M., Wieland M., Geis-Gerstorfer J. (2006). Enhancing surface free energy and hydrophilicity through chemical modification of microstructured titanium implant surfaces. J. Biomed. Mater. Res. A.

[26] Klein M.O., Bijelic A., Ziebart T., Koch F., Kammerer P.W., Wieland M., Konerding M.A., Al-Nawas B. (2013). Submicron Scale-Structured Hydrophilic Titanium Surfaces Promote Early Osteogenic Gene Response for Cell Adhesion and Cell Differentiation. Clin. Implant Dent. Relat. Res..

[27] Su Y., Luo C., Zhang Z., Hermawan H., Zhu D., Huang J., Liang Y., Li G., Ren L. (2018). Bioinspired surface functionalization of metallic biomaterials. J. Mech. Behav. Biomed..

[28] Wong H.W., Zhao Y., Leung F.K.L., Xi T., Zhang Z., Zheng Y., Wu S., Luk K.D.K., Cheung K.M.C., Chu P. (2017). Functionalized Polymeric Membrane with Enhanced Mechanical and Biological Properties to Control the Degradation of Magnesium Alloy. Adv. Healthc. Mater..

[29] Li Z., Liu W., Yang H., Sun T., Liu K., Wang Z., Niu C. (2015). Improved thermal dehydrogenation of ammonia borane by MOF-5. RSC Adv..

[30] Rocca J.D., Liu D., Lin W. (2011). Nanoscale Metal-Organic Frameworks for Biomedical Imaging and Drug Delivery. Acc. Chem. Res..

[31] Horcajada P., Gref R., Baati T., Allan P.K., Maurin G., Couvreur P., Férey G., Morris R.E., Serre C. (2012). Metal-Organic Frameworks in Biomedicine. Chem. Rev..

[32] Hu Q., Yu J., Liu M., Liu A., Dou Z., Yang Y. (2014). A Low Cytotoxic Cationic Metal-Organic Framework Carrier for Controllable Drug Release. J. Med. Chem..

[33] Mesbah A., Jacques S., Rocca E., Francois M., Steinmetz J. (2011). Compact Metal-Organic Frameworks for Anti-Corrosion Applications: New Binary Linear Saturated Carboxylates of Zinc. Eur. J. Inorg. Chem..

[34] Wu C., Liu Q., Chen R., Liu J., Zhang H., Li R., Takahashi K., Liu P., Wang J. (2017). Fabrication of ZIF-8@SiO_2_ Micro/Nano Hierarchical Superhydrophobic Surface on AZ31 Magnesium Alloy with Impressive Corrosion Resistance and Abrasion Resistance. ACS Appl. Mater. Interfaces.

[35] Bernini M.C., Fairen-Jimenez D., Pasinetti M., Ramirez-Pastor A.J., Snurr R.Q. (2014). Screening of bio-compatible metal-organic frameworks as potential drug carriers using Monte Carlo simulations. J. Mater. Chem. B.

[36] Zhang C.Y., Zeng R.C., Liu C.L., Gao J.C. (2010). Comparison of calcium phosphate coatings on Mg-Al and Mg-Ca alloys and their corrosion behavior in Hank’s solution. Surf. Coat. Technol..

[37] Ben T., Lu C., Pei C., Xu S., Qiu S. (2012). Polymer-Supported and Free-Standing Metal-Organic Framework Membrane. Chem. Eur. J..

[38] Kokubo T., Takadama H. (2006). How useful is SBF in predicting in vivo bone bioactivity?. Biomaterials.

[39] Guo H., Zhu G., Hewitt I.J., Qiu S. (2009). “Twin Copper Source” Growth of Metal-Organic Framework Membrane: Cu_3_(BTC)_2_ with High Permeability and Selectivity for Recycling H_2_. J. Am. Chem. Soc..

[40] Liu J., Sun F.X., Zhang F., Wang Z., Zhang R., Wang C., Qiu S.L. (2011). In situ growth of continuous thin metal-organic framework film for capacitive humidity sensing. J. Mater. Chem..

[41] Caskey S.R., Wong-Foy A.G., Matzger A.J. (2008). Dramatic Tuning of Carbon Dioxide Uptake via Metal Substitution in a Coordination polymer with Cylindrical Pores. J. Am. Chem. Soc..

[42] Zhang F.Z., Zhao L.L., Chen H.Y., Xu S.L., Evans D.G., Duan X. (2008). Corrosion resistance of superhydrophobic layered double hydroxide films on aluminum. Angew. Chem. Int. Ed..

[43] Park J.H., Schwartz Z., Olivares-Navarrete R., Boyan B., Tannenbaum R. (2011). Enhancement of Surface Wettability via the Modification of Microtextured Titanium Implant Surfaces with Polyelectrolytes. Langmuir.

[44] Martines E., Seunarine K., Morgan H., Gadegaard N., Wilkinson C.D.W., Riehle M.O. (2005). Superhydrophobicity and Superhydrophilicity of Regular Nanopatterns. Nano Lett..

[45] Yang J., Morelock C.R., Burtch N.C., Mounfield W.P., Hungerford J.T., Walton K.S. (2015). Tuning the Kinetic Water Stability and Adsorption Interactions of Mg-MOF-74 by Partial Substitution with Co or Ni. Ind. Eng. Chem. Res..

